# Methicillin-Resistant *Staphylococcus aureus* in Saarland, Germany: The Long-Term Care Facility Study

**DOI:** 10.1371/journal.pone.0153030

**Published:** 2016-04-13

**Authors:** Dorothea Nillius, Lutz von Müller, Stefan Wagenpfeil, Renate Klein, Mathias Herrmann

**Affiliations:** 1 Institute and State Laboratory of Medical Microbiology and Hygiene, Saarland University and Saarland University Medical Centre, Homburg, Germany; 2 Institute of Medical Biometry, Epidemiology, and Medical Informatics, Saarland University, Homburg, Germany; 3 Saarland Ministry of Social Affairs, Health, Women, and Family, Saarbrücken, Germany; Universitätsklinikum Hamburg-Eppendorf, GERMANY

## Abstract

**Background:**

Multiresistant organisms pose a threat for patients and care recipients. Control interventions need to be tailored to region, the type of institution considered, and risk factors. The German state of Saarland is ideally suited to study colonisation epidemiology throughout its various health and care institutions. After conclusion of a large admission prevalence study in acute care hospitals, we now performed a methicillin-resistant *Staphylococcus aureus* (MRSA) point prevalence study in Saarland long term care facilities (LTCF), allowing for a direct comparison with respect of MRSA prevalence and associated risk factors between these two institutional types located within a confined region.

**Methodology and Principal Findings:**

Of all LTCF of the region, 65/136 participated in the study performed between 09/2013 and 07/2014. Overall, complete microbiological specimen and questionnaires of 2,858 of 4,275 (66.8%) LTCF residents were obtained. 136/2,858 (4.8%) screened residents revealed MRSA carrier status. Multivariate risk factor analysis yielded ulcer/deep soft tissue infection, urinary tract catheter, and MRSA history with multiple MRSA decolonisation cycles to be independently associated with MRSA carrier status.

**Conclusion:**

As already known from previous studies, colonisation with MRSA is common in LTCF residents even in an area with relatively low MRSA prevalence. This found prevalence can now be related to the acute care admission prevalence (2.2%) as well as to the admission prevalence in acute care geriatric departments (7.6%). The common clonal attribution (*spa* type) of MRSA isolates prevalent in the LTCF population as well as in the acute care admission population points towards a close relationship between both types of institutions. However, the ostensible absence of risk factors such as “previous hospitalisation” in conjunction with newly identified factors such as “multiple decolonisation cycles” refers to MRSA colonisation risks independent of contact with acute care facilities. Overall, this large LTCF point prevalence study allows data-based, region-tailored decisions on MRSA screening policies and provides a basis for additional preventative measures.

## Introduction

The state of Saarland is Germany’s smallest non-city federal state, an administrative entity ideally suited for state-wide comprehensive and comparative analyses on the prevalence of methicillin-resistant *Staphylococcus aureus* (MRSA) in various types of (health) care institutions (traditionally, Saarland hosts various health registries, and the 'MRSAar net' (please refer below) provides contact to all acute and chronic care institutions). In 2013, the first German state-wide study on the prevalence of MRSA in hospital admission patients (subsequently referred to as the ‘admission prevalence study’, AP study) has been concluded [1], and its results reporting from all Saarland acute care institutions are deemed to representatively reflect the current state of MRSA burden in the region and beyond [2, 3]. In this study, a prevalence of 2.2 MRSA carriers per 100 hospital admissions was reported, the risk for MRSA carriage could be associated with defined clinical and patient history factors, and conclusions from this study are incorporated in the current German recommendations for prevention and control of MRSA in medical facilities [4].

MRSA colonisation significantly contributes to ensuing infection, often after prolonged intervals of carrier status [5, 6] adding to enhanced morbidity. These infections add to the overall infection rate by *S*. *aureus*, and do not replace infections to methicillin-sensitive isolates [7, 8]. Crude *S*. *aureus* bloodstream infection mortality rates are clearly elevated if the infection is caused by a methicillin-resistant compared to a methicillin-sensitive isolates [9–12], a fact likely owed to inadequate initial therapy [13, 14] albeit also confounded by comorbidity [14]. Thus, nosocomial MRSA continues to be endemic in hospitals and long-term care facilities (LTCF) overall in Europe causing added burden of disease [15–18]. Moreover, it is generally acknowledged that patient transfer and associated isolate spread between acute care facilities and LTCF contribute to the continued MRSA endemic of all parts of the health care system [19–22]. Thus, as part of the analytic and intervening strategy of the ‘Regional Network on Prevention and Control of MRSA in Saarland’ (www.mrsaar.net), in this study the prevalence of MRSA in Saarland LTCF has been analysed. Moreover, risk factors for MRSA colonisation in LTCF inhabitants differ from those associated with MRSA carriage in hospital admission patients [1], thus, a detailed analysis of such risk factors among LTCF inhabitants was also performed.

## Material and Methods

### Microbiological Analyses

In order to exclude any bias due to inter-laboratory variabilities, all samples were processed in the medical microbiology diagnostic facility of the authors’ institution (Institute of Medical Microbiology and Hygiene at the Saarland University Medical Centre). Samples were processed using an automated system, the Walk Away Specimen Processor, WASP (Copan, Brescia, Italy) [23, 24]. Although the overall study was executed from 09/2013 to 07/2014, the sampling and data acquisition period in each facility lasted for a maximum of three days. Residents which choose to participate, but were absent during these day (e.g. due to an admission in a hospital or another relocation) were not included in the study. The swabs were carried out by the LTCF personnel on the basis of a detailed and illustrated description. In brief, these instructions detailed the swabbing of the *vestibulum nasi* of both nares with one swab (ESwab, Copan), then transfer of the swab into the transport medium. This method is in line with a previous analysis [25] and acknowledges the recently published results for optimal swab selection for this purpose [26]. A flocked swab system was employed, based on a liquid specimen microbiology technique and allowing for immediate release of swabbed microorganisms into the Amies medium. One of the swabs was used for the pharyngeal site and a second for both anterior nares. The first swab was swivelled in the elution medium then discarded; the second swab was immersed in the same elution medium and remained there until processing in the laboratory. Specimens were directly plated on CHROMagar MRSA detection biplates (Mast, Germany) for the simultaneous detection of MRSA and methicillin-sensitive *S*. *aureus* (MSSA) as reported elsewhere [1]. All MRSA positive culture isolates were further confirmed using a species identification by mass spectrometry (MALDI Microflex, Bruker, Germany), penicillin binding-protein 2a test (Alere, Germany), latex agglutination test (Pastorex Staph-Plus, BioRad, Germany), resistogram (Vitek, bioMérieux, USA, and EUCAST criteria), and further subjected to *spa* typing as previously described [27]. Genetic tests for the *mecA* gene as well as a specific fragment of the *pvl* (Panton-Valentine leucocidin) gene, were performed using GenoType Staphylococcus Test according to instructions of the manufacturer (HAIN Lifescience, Nehren, Germany). No discrepant results between phenotypic results for methicillin resistance (growth on MRSA selective agar, antimicrobial susceptibility testing, PBP2a test) and a positive molecular test for the *mecA* gene were observed. In line with Becker et al. [28], for these *mecA* positive isolates we refrained from *mecC* testing.

### Region

The State of Saarland is located in the southwest of Germany and is neighboured by France, Luxembourg, and the German State of Rhineland-Palatinate. It currently comprises of 997,855 inhabitants, with 10,407 of residents residing in 136 LTCF [29].

### Study Period, LTCF Participation

All 136 LTCF of the region were invited for participation, 65 (48%) participated in the study from 09/2013 to 07/2014 (nursing homes for the elderly as well as foster homes for handicapped people). We obtained only limited information from LTCF which were invited for participation but refused. The institutions which provided us with an explanation for non-participation mostly cited a lack of personnel qualified in obtaining the LTCF resident data and performing the necessary microbiologic specimen acquisition. Moreover, we obtained no detailed information on the non-participating residents’ biographic or medical data; thus, a potential bias due to non-participation with respect of the entirety of Saarland LTCF cannot be entirely excluded. The overall structure of these non-participating LTCF, however, is not different from those institutions participating in the study. 56 of the participating 65 LTCF provided complete reports on occupancies or patient characteristics. According to these available data, a participation rate of less than 25%, 25–50%, 50–75%, and more than 75% of residents was observed in five, 16, 28, and 16 LTCF, respectively.

The only study inclusion criterion was residence in a participating LTCF during the study period; no exclusion criteria were employed. Within institutions, each participating resident received a pseudonym identifier. Consecutive labelling of questionnaire and swabs made inadvertent double sampling impossible. Potentially, double sampling could be possible if one resident would have been transferred between institutions; however, this is also highly unlikely as residents typically conclude long term care and rental contracts with the respective institution, and a transfer between institutions is a rare exception. The pseudonym identifier also allowed the LTCF administration to allocate positive MRSA results and to provide for antiseptic material kits and decolonisation measures for their residents.

### Study Questionnaire

For each resident, institutions were asked to complete a risk factor evaluation form providing the pseudonym identifier. These questionnaires were completed by the LTCF nursing staff based on patient records, written information contained a description on how fill in the questionnaires. Data collection included patient-related data and demographic characteristics, functional status, comorbid conditions, underlying diseases, presence of indwelling devices, nasogastric feeding, previous hospitalisation, and antibiotic exposure (described in Table 1). According to the German Social Security Act XI, the degree of dependency of care (personal care, nutrition, mobility) is described by care levels, with care level I defined as significant, and level III as heavy or full dependency. Notably, residents with dementia, psychologically or physically disabled persons with reduced everyday competence yet not meeting the formal care level criteria are classified as care level 0.

**Table 1:** Risk factor distribution of the study population and MRSA carriers.

Risk factor	Study population n / N	MRSA carriers N / N
single room accommodation	1,361 / 2,858 (47.6%)	64 / 136 (47.1%)
shared room accommodation	1,442 / 2,858 (50.5%)	67 / 136 (49.3%)
PEG tube	137 / 2,831 (4.8%)	18 / 136 (13.2%)
animal (pet)	509 / 2,858 (17.8%)	18 / 136 (13.2%)
urinary incontinence	1,723 / 2,858 (60.3%)	78 / 136 (57.4%)
urinary tract catheter (UTC)	350 / 2,858 (12.2%)	40 / 136 (29.4%)
dialysis catheter	13 / 2,858 (0.5%)	1 / 136 (0.7%)
impairment of skin barrier^1^	646 / 2,858 (22.6%)	59 / 136 (43.4%)
decubitus	70 / 2,858 (2.4%)	9 / 136 (6.6%)
diarrhoea^#^	100 / 2,858 (3.5%)	6 / 136 (4.4%)
infection^2^ ^#^	597 / 2,858 (20.9%)	40 / 136 (29.4%)
infection of gastrointestinal tract	36 / 2,858 (1.3%)	2 / 136 (1.5%)
infection of skin / deep soft tissue	100 / 2,858 (3.5%)	6 / 136 (4.4%)
infection of the respiratory tract	281 / 2,858 (9.8%)	11 / 136 (8.1%)
urogenital infection / UTI	140 / 2,858 (4.9%)	19 / 136 (14.0%)
infection of moth / nose / eyes / ears	57 / 2,858 (2.0%)	2 / 136 (1.5%)
stoma (total)	78 / 2,858 (2.7%)	7 / 136 (5.1%)
tracheostomy	19 / 2,858 (0.7%)	2 / 136 (1.5%)
ileostomy	32 / 2,858 (1.1%)	4 / 136 (2.9%)
gastrostomy	31 / 2,858 (1.1%)	1 / 136 (0.7%)
COPD	229 / 2,858 (8.0%)	12 / 136 (8.8%)
vascular disease	877 / 2,858 (30.7%)	48 / 136 (35.3%)
autoimmune disease	32 / 2,858 (1.1%)	0 / 136 (0%)
diabetes	682 / 2,858 (23.9%)	37 / 136 (27.2%)
cancer	186 / 2,858 (6.5%)	12 / 136 (8.8%)
immunosuppression	12 / 2,858 (0.4%)	0 / 136 (0%)
antacid use	1,241 / 2,858 (43.4%)	56 / 136 (41.2%)
contact with MRSA carrier (shared accommodation)	26 / 2,858 (0.9%)	5 / 136 (3.7%)
antibiotics^#^	506 / 2,858 (17.7%)	27 / 136 (19.9%)
fluoroquinolone^#^	150 / 2,858 (5.2%)	16 / 136 (11.8%)
hospitalisation (> 3d^#^)	321 / 2,858 (11.2%)	19 / 136 (14%)
history of MRSA colonisation	128 / 2,858 (4.5%)	30 / 136 (22.1%)
performed multiple MRSA decolonisation cycles	48 / 121 (39.7%)	17 / 29 (58.6%)
previous intestinal MDRO carriage	62 / 2,858 (2.2%)	7 / 136 (5.1%)
previous *C*. *difficile*	29 / 2,858 (1.0%)	2 / 136 (1.5%)

^1^ stoma, dialysis catheter, urinary tract catheter, gangrene, necrosis, chronic wound, ulcer, decubitus, skin / soft tissue infection, PEG tube, parenteral nutrition

^2^ infection of gastrointestinal / urogenital / respiratory tract, skin, eye, ear, mouth, nose, or deep tissue

^#^ within a period of three months

n / N: number of residents with risk factors / number of residents with risk factors evaluable

The Commission for Hospital Hygiene and Infection Prevention at the Robert Koch Institute (RKI) (Berlin) has issued a list of risk factors recommended for risk-adapted screening purposes [30] which delineate the targeted MRSA screening requirements for acute care facilities according to the German ‘Infection Protection Act’. These risk factors were also evaluated in the questionnaire.

### Ethics Statement

This study was approved by the Ethics Committee at the Chamber of Physicians of the State of Saarland (127/10) and by the Saarland State Commissioner for Data Protection. Written informed consent was obtained from all participants or their legal representatives.

### Statistical Methods

For categorical variables, all figures are reported as absolute or relative frequencies. Risk analysis was performed using binary logistic regression. Pooled prevalence rates were calculated according to a random effects model to account for possible cluster effects with respect to LTCF. Any p-values given are two-sided and subject to a significance level of 0.05 as well as odds ratios (OR) with 95% confidence intervals according to Pearson-Mantel-Haenszel. Group differences in relative frequencies of MRSA positive rates are due to Fisher's exact test. All analyses were done using IBM SPSS Version 22 and StatsDirect statistical software Version 3.0.155.

## Results

### Study Population

For the study period, the participating nursing homes reported to have 5,234 care places, 4,275 of them occupied. All of their residents were invited for participation. We received 2,942 resident materials, owing to a participation rate of 68.8%. Of these sets, 2,858 were complete with swabs and questionnaire (97.1%), 20 (0.7%) were samples without fully evaluable questionnaire and 64 were (2.2%) questionnaires only.

### Age, Gender, and Nursing Characteristics

For 2,820/2,858 residents of the study population, the year of birth was available. The mean age of the study population was 81.5 years (SD: 11.7 years), the median age was 84 years (25^th^ and 75^th^ percentile, 76 and 90 years, respectively). Additional demographic characteristics are shown in Table 1. For 2,823/2,858 residents, valid gender identification was available; 2,004 (71%) of them were females. We found 1,466 (56.2%) residents to suffer from dementia. Care levels were attributed to the residents as follows: 312 (11.1%) for level 0, 1,149 (40.8%) for level I, 931 (33.0%) for level II, and 427 (15.1%) for level III. 714 (25.2%) residents were autonomous in their activity, 741 (26.2%) independent with a walking aid, 963 (34.0%) had a restricted activity level (wheel chair), 39 (1.4%) were bedridden but with a possible change of position, and 376 (13.3%) were bedridden and completely dependent from others.

### *Staphylococcus aureus* and MRSA Prevalence

**Fig 1:**
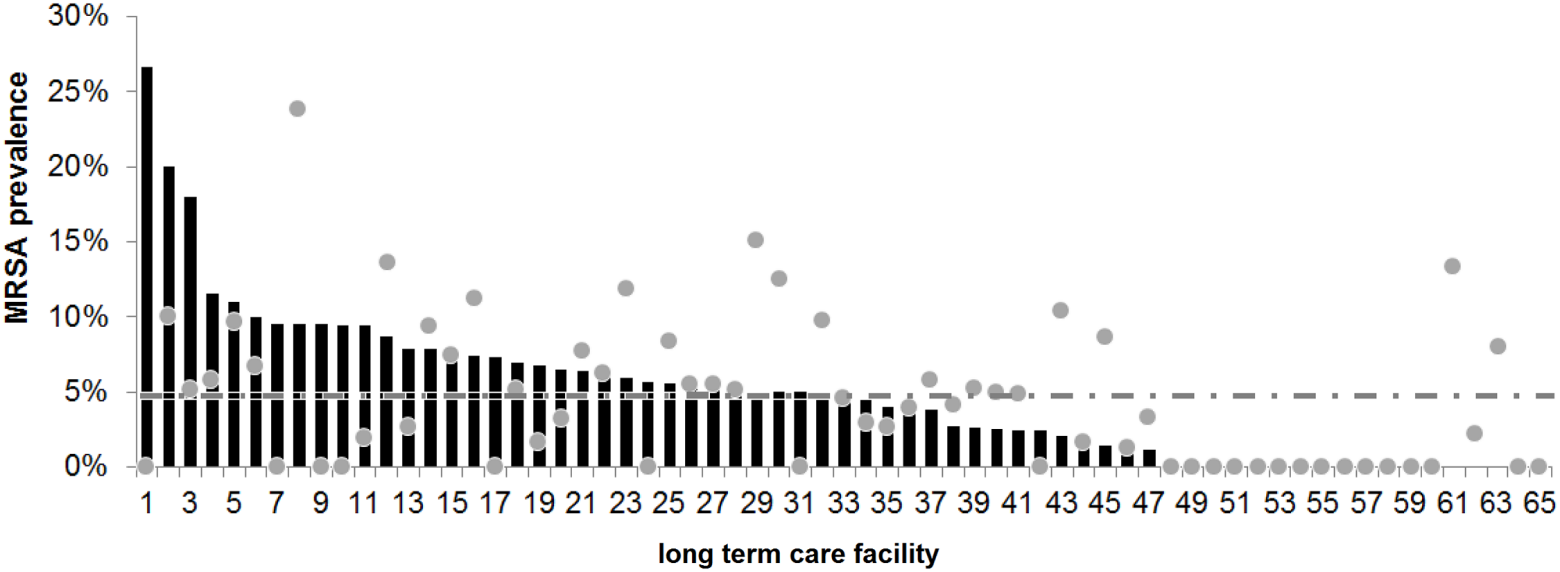
MRSA prevalence in nursing homes in Saarland, Germany. Shown is the MRSA prevalence (MRSA cases in percent) of the various LTCF sorted by result. The dots represent the expected rate of MRSA prevalence based to the LTCF pre-study information; the dashed line shows the mean MRSA rate throughout the entire study population.

Altogether, 940/2,858, i.e. 32.9% of the residents were found to be positive in nares / pharynx for *S*. *aureus*. Of these, 136 were found to be positive for MRSA, corresponding to an overall prevalence of 4.76% for MRSA and 28.13% for MSSA, respectively. The mean overall MRSA prevalence between participating nursing homes was 4.89% (CI_95_, 3.87–5.92%, pooled proportion using random effects (DerSimonian-Laird), range 0% to 26.7%) (Fig 1).

None of the tested isolates was found to be *pvl* positive.

### *S*. *aureus* Protein A (*spa*) Typing

**Fig 2:**
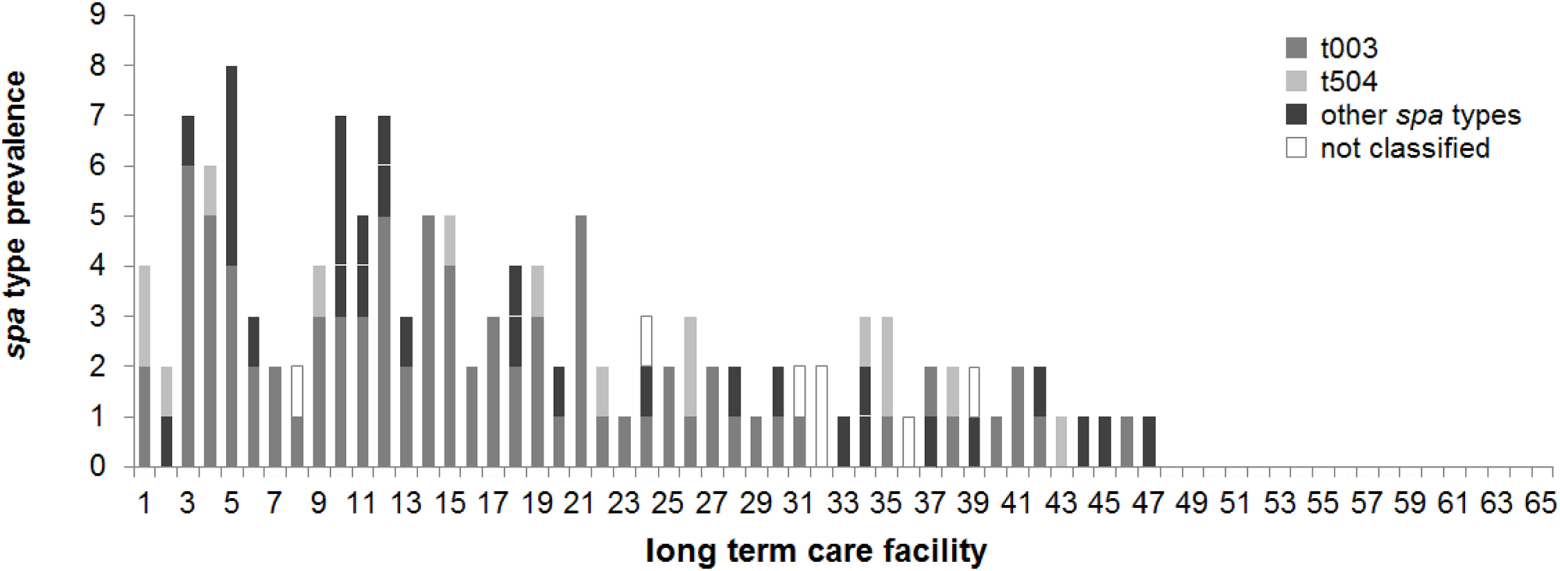
Prevalence of *spa* types in nursing homes in Saarland, Germany. The number of different *spa* types is given per LTCF (identical numbering as in Fig 1). *spa* type t003 (grey), t504 (light grey), not classified *spa* types (white), other *spa* types (black).

A total of 129/136 MRSA isolates was available for *spa* typing. 83 (64.3%) of these isolates were attributable to *spa* sequence type t003 (corresponding to ST5/CC5, the Rhine-Hesse/EMRSA-3/New York clone), 15 (11.6%) to t504 (closely related to t003), and 4 (3.1%) to t458. Three isolates (2.3%) each belonged to t004, t008, t020, and t476, respectively, and two isolates (1.5%) each to t002, t045, and t463, respectively. Another ten *spa* types occurred only once (Fig 2).

### Risk Factor Analysis

Only 2/136 (1.5%) of all MRSA colonised residents did not report any risk factor. The absolute and relative presence of risk factors both in the entire study population as well as in the MRSA positive study cohort is shown in Table 1.

Univariate analysis revealed that a number of risk factors were found to be associated with a significantly increased MRSA positivity (Table 2). The highest probability for MRSA carriage—increasing the risk of MRSA colonisation by a factor of more than seven—was ‘history of MRSA colonisation’; moreover, the previous contact with an MRSA carrier, e.g. by sharing the same room, enhanced the risk almost fivefold. Significantly associated with an MRSA carrier status albeit with lower OR is the presence of urogenital conditions such as an urinary tract catheter or a history of a urogenital infection / UTI, physical inactivity (bedridden; change of position possible), an elevated care level (but not the complete care dependence level III), or various categories of impaired skin conditions such as decubitus, ulcer, other chronic wound or other skin barrier alterations. Male residents carried an almost double risk for MRSA carriage when compared with female residents. Finally, while infection of any site was associated with a moderately increased MRSA carriage risk, also the concurrent intestinal colonisation with multi drug resistant organisms (MDRO) like gramnegative rods or vancomycin-resistant enterococci was associated with MRSA colonisation. (A manuscript on the data on the prevalence of MDRO in LTCF in Saarland is currently in preparation).

**Table 2:** Univariate analysis of risk factors associated with MRSA colonisation.

Risk Factor	n / N	OR (95% CI); p-value
history of MRSA carriage	30 / 2,858	7.58 (4.82–11.92); <0.001**
contact with MRSA carrier	5 / 2,858	4.91 (1.82–13.22); 0.002**
physical activity: bedridden, change of position is possible^1^	4 / 2,833	3.97 (1.29–12.23); 0.016*
urogenital infection / UTI	19 / 2,858	3.49 (2.08–5.86); <0.001**
nutrition: PEG tube^1^	18 / 2831	3.30 (1.91–5.59); <0.001**
urinary tract catheter (UTC)	40 / 2,858	3.24 (2.20–4.78); <0.001**
decubitus	9 / 2,858	3.09 (1.5–6.36); 0.002**
ulcer / deep soft tissue infection	5 / 2,858	2.93 (1.13–7.60); 0.027*
ileostomy	4 / 2,858	2.92 (1.01–8.43); 0.048*
multiple MRSA decolonisation cycles	17 / 2,858	2.79 (1.18–6.56); 0.019*
skin barrier^2^	59 / 2,858	2.79 (1.96–3.96); <0.001**
fluoroquinolone	16 / 2,858	2.58 (1.49–4.46);0.001**
chronic wound	8 / 2,858	2.44 (1.15–5.18); 0.020*
physical activity: limited / wheel chair^1^	59 / 2,833	2.27 (1.35–3.80); 0.002**
physical activity: bedridden, completely dependent on help^1^	21 / 2,833	2.05 (1.10–3.84); 0.024*
male gender	59 / 2,823	1.94 (1.37–2.76); <0.001**
infection^3^	40 / 2,858	1.62 (1.11–2.37); 0.013*
care level II	57 / 2,819	1.53 (1.08–2.18); 0.017*
nourishment	0.336^#^	1.4 (1.17–1.68); <0.001**
physical activity	0.189^#^	1.21 (1.06–1.38); 0.005**

Risk factors are ranked according to OR’s of univariate analysis. Only significant risk factors depicted in the table (** highly significant with p<0.01, * significant with p<0.05).

^1^ For categorical data (care level, nutrition, and physical activity) dummy coding was used.

^2^ impairment of skin barrier (stoma, dialysis catheter, urinary tract catheter, gangrene, necrosis, chronic wound, ulcer, decubitus, skin / soft tissue infection, PEG tube, parenteral nutrition)

^3^ infection of gastrointestinal / urogenital / respiratory tract, skin, eye, ear, mouth, nose, or deep tissue

^#^ regression coefficient

n / N: number of MRSA positive residents with risk factors identified / number of residents with risk factors evaluable

We asked for the use of antibiotics in the last three months and the selected substance groups (penicillins, cephalosporins, carbapenems, macrolides, tetracycline, trimethoprim, clindamycin, fluoroquinolones). Interestingly, the general use of antibiotic is not a risk for MRSA colonisation, but in contrast fluoroquinolone use was associated with a more than twofold enhanced MRSA risk in univariate analysis.

MRSA colonised residents were not of significantly different age compared to the overall study population (mean age 80.4 ± 12.0 years vs. 81.5 ± 11.7 years in the overall study population). In the comparably small group of chronic care residents with 30–39 years of age, the MRSA prevalence was 2/11 (18.2%), while in other age groups the MRSA prevalence varied between 0% and 5.8%. Using a logistic regression approach, a trend for an inverse correlation between age and prevalence could be demonstrated: the older the LTCF residents were, the rarer a MRSA colonisation was demonstrable. In contrast, when examining the male population only, an opposite trend could be confirmed with enhanced colonisation as a function of increased age. Both trends, however, did not reach significance level.

A significant correlation was found between MRSA colonisation and care dependency during food uptake. Moreover, in the resident group with a PEG tube a disproportionately high MRSA rate was observed (4.8% of all residents vs. 13.2% of MRSA carriers), leading to a more than threefold increased risk. When comparing MRSA carrier status with physical activity, it was found that reduced physical activity and autonomy was associated with increased likelihood of MRSA carriage.

In contrast, neither a recent hospital stay of more than three days, nor diabetes or dementia were associated with a significantly enhanced likelihood of a MRSA carriage. The size of the care facility had also no effect on the colonisation rate.

Interestingly, when compared to the relatively large list of risk factors significantly associated with MRSA colonisation in the univariate analysis, in multivariate analysis using conditional logistic regression, only three factors were significantly associated with an increased risk, i.e. ‘ulcer / deep soft tissue infection‘, ‘urogenital tract catheter’ (each associated with a more than fivefold risk), and ‘multiple MRSA decolonisation cycles’ (almost threefold risk) (Table 3).

**Table 3:** Multivariate analysis of risk factors associated with MRSA colonisation.

Risk factor	OR (95% CI); p-value
ulcer / deep soft tissue infection	6.61 (1.14–38.37); 0.035*
urinary tract catheter (UTC)	5.21 (1.84–14.76); 0.002**
multiple MRSA decolonisation cycles	2.79 (1.02–7.64); 0.046*

For multivariate analysis (logistic regression; backward; Wald), all univariate risk factors were used with the exception of ‘skin barrier’, ‘infection’, and results of rectal swabs (concomitant intestinal carriage of MDRO).

Only significant risk factors depicted (** highly significant with p<0.01, * significant with p<0.05).

### Risk Factor-based Screening Approach

This study allows calculating the number of LTCF residents to be screened for identification of MRSA carriers as a function the identified risk factors. Such calculation was performed by sorting the rank of significant OR results in the risk analysis, beginning with the risk factor associated with highest OR. The number of cumulative risk factor entries was assessed by stepwise inclusion of the subsequent factors, and the relative proportion of residents with the respective risk factor or risk factor combination was expressed in percent. In parallel, the respective number of residents shown to be positive for MRSA carrier status was determined. Finally, a ‘ratio’ describing the number of residents to be screened in order to detect one MRSA carrier was calculated (Table 4).

**Table 4:** Cumulative proportions of patients reporting one or several risk factors and resulting number-to-screen.

**Based on multivariate risk factors (this study)**	**risk factor entry**	**MRSA detected**	**ratio**^#^
ulcer / deep soft tissue infection	40 (1.4%)	5 (3.7%)	8.0
urinary tract catheter (UTC)	350 (8.8%)	40 (12.3%)	8.8
ulcer + urinary tract catheter (UTC)	386 (13.5%)	44 (32.4%)	8.8
ulcer + UTC + multiple decolonisation cycles	414 (14.5%)	52 (38.2%)	8.0
**Based on univariate risk factors (this study)**	**risk factor entry**	**MRSA detected**	**ratio**^#^
history of MRSA carriage	128 (4.5%)	30 (22.1%)	4.3
+ contact with MRSA carrier	150 (5.2%)	32 (23.5%)	4.7
+ physical activity: bedridden, change of position is possible	184 (6.4%)	35 (25.7%)	5.3
+ urogenital infection / UTI	305 (10.7%)	48 (35.3%)	6.4
+ nutrition: PEG tube	399 (14.0%)	53 (39.0%)	7.5
+ urinary tract catheter (UTC)	604 (21.1%)	65 (47.8%)	9.3
+ decubitus	629 (22.0%)	65 (47.8%)	9.7
+ ulcer / deep soft tissue infection	656 (23.0%)	67 (49.3%)	9.8
+ ileostomy	678 (23.7%)	69 (50.7%)	9.8
+ multiple MRSA decolonisation cycles	678 (23.7%)	69 (50.7%)	9.8
+ skin barrier	801 (28.0%)	73 (53.7%)	11.0
+ fluoroquinolone	863 (30.2%)	76 (55.9%)	11.4
+ chronic wound	863 (30.2%)	76 (55.9%)	11.4
+ physical activity: limited / wheel chair	1493 (52.2%)	103 (75.7%)	14.5
+ physical activity: bedridden, completely dependent on help	1643 (57.5%)	106 (77.9%)	15.5
+ male gender	1998 (69.9%)	116 (85.3%)	17.2
+ infection	2074 (72.6%)	116 (85.3%)	17.9
+ care level II	2210 (77.3%)	120 (88.2%)	18.4
**Based on risk factors (AP study; recommendations of the Robert Koch Institute)**	**risk factor entry**	**MRSA detected**	**ratio**^#^
AP study [1]	2188 (76.6%)	116 (85.3%)	18.8
RKI 2014 [30]	1141 (39.9%)	74 (54.4%)	15.4
RKI 2005 [31]	2378 (83.5%)	118 (86.8%)	20.1
RKI 1999 [32]	1859 (65.0%)	105 (77.2%)	17.7

^#^ The ratio describes the number of residents to be screened in order to detect one MRSA carrier.

Based on the univariate risk factor list, and applying the factor with highest OR (‘history of MRSA carriage’), 4.5% of the residents would have to be screened, and such screening would yield a detection of 22.1% MRSA carriers. If all (univariate) risk factors would be applied, 77.3% of the resident population would be needed to be screened in order to detect 88.2% of MRSA. If the risk factors identified in the multivariate analysis would be used, the risk factor combination ‘ulcer’, ‘urinary tract catheter’ and ‘multiple decolonisations’ would allocate 14.5% of the resident population to be screened while unveiling 38.2% of residents with MRSA carrier status. In this setting, eight residents would be needed to be screened to detect one MRSA carrier. Finally, we have calculated the respective proportions based on the risk factors either identified in the admission prevalence studies of Saarland acute hospitals as well as based on various recommendations issued by the RKI (risk factors from these recommendations and applying to every resident of LTCF, i.e. ‘nursing home‘, ‘old age‘, and ‘dependency‘, were excluded).

## Discussion

To our knowledge, this is the second-largest MRSA point-prevalence survey ever performed in LTCF; only one study [33] almost 15 years ago having investigated a slightly larger number of LTCF residents. The participation rate of 68.8% of residents living in participating LTCF at the time of the point prevalence study is also clearly larger than participation rates reported in other studies (e.g. the recent Rhine-Main district study reporting a participation rate of 29% [34]).

Overall, in this study 4.8% LTCF residents were found to be MRSA positive. This MRSA prevalence rate in LTCF residents is inferior to rates reported in previous LTCF studies from Germany such as one from the Frankfurt/Main area (9.0%, 9.2%, and 6.2% in studies from 2006, 2012, and 2013, respectively) [34–36] or Brunswick (7.6% in 2009) [37], but it is clearly higher than reported in a study from North Rhine-Westphalia (3%) [38] and from the Rhine-Neckar region (1.1%) [33] (both studies from year 2000). Compared to international studies, the MRSA prevalence rate in our study was also found to be inferior than reported in studies from the USA (24% 1998, 6.3% 2003, and 31% in 2008, respectively) [39–41], from Hong Kong (21.6% in 2011) [42], or Luxembourg (7.2% in 2010) [43], but larger than in geriatric and/or rehabilitative wards of hospitals in the Dutch-German border area (2.6% in 2006) [44], in the Netherlands (0.3% in 2009) [45], or in Sweden (0% in 2012) [46].

As an indicator of the preanalytical and analytical detection quality, the prevalence for *S*. *aureus* (MRSA and MSSA isolates combined) in this study was found to be 32.9%, a figure that closely reflects the *S*. *aureus* prevalence reported in other studies in adult populations analysed for MRSA prevalence purposes [44, 47, 48]. This figure allows to compare the fraction of MRSA/MSSA (frequently referred to as “MRSA rate”) in our study with those rates previously reported: here we determined 14.5% of the total *S*. *aureus* strains to be methicillin resistant, corresponding to a ratio of approximately one MRSA positive resident for every seven MSSA positive residents.

To our knowledge, this is the only large LTCF MRSA prevalence study analysing in parallel the prevalence of MSSA, thus, broader comparisons of the here calculated MRSA rates with other LTCF point prevalence studies are not feasible. One smaller study from 2002 in nursing homes in North Rhine-Westphalia revealed one MRSA positive for approximately every 16 *S*. *aureus* positive residents (MRSA rate 6.3%) [38]. Interestingly, findings from acute care institutions (e.g., the German counties participating in the Euregio study [44]) detected 6.5% of methicillin resistant isolates, while the AP study in Saarland [1] revealed a rate of 14.0%. Such elevated MRSA rates in the LTCF population appear plausible, because nursing home residents are associated with at least one risk factor for MRSA colonisation, i.e. chronic care [30–32].

*Spa* typing identified a total of 20 different *spa* types, with t003 being the most frequently represented type, and t504 genotype also being relatively frequent; the latter being a very typical regional strain closely related to the predominant t003 [49]. Both *spa* types encomprise 76% of the MRSA isolates recovered while other *spa* types were only sparsely encountered. This quite exactly reflects the results from the AP study indicating 75% of all MRSA isolates belonging to these two *spa* types t003 and the ‘Saarland’ type t504, suggestive of a tight relationship between the MRSA populations of both LTCF residents and acute care hospital admission patients. The Rhine-Hesse clone t003 was also the most prevalent *spa* type in nursing homes in Frankfurt/Main [34–36], in patients of an acute care and rehabilitation clinic in Siegen-Wittgenstein (North Rhine-Westphalia) [47], or in residents of LTCF in Luxembourg [43]. Interestingly, isolates of t022 *spa* type (corresponding to CC22, or UK EMRSA-15 clone) were not detected in our population while approximately ⅓ of the isolates of the most recent Rhine-Main study [34] belonged to this *spa* type.

Of further interest is the finding that no community acquired ca-MRSA (PVL positive) or livestock associated la-MRSA was identified (; none of the *spa* types found in this study belonged to any of the livestock-associated *spa* types as described by Hetem et al. [50]). This is in concordance with the AP study [1] and other surveys in LTCF in Germany [15, 36].

In multivariate analysis, the presence of an ulcer / deep soft tissue infection constitutes the highest risk for MRSA carrier status (OR 6.6). This is clearly elevated compared with risk factor findings in a LTCF population in Belgium (decubitus and skin ulcer: OR 2.6) [51], in the AP study (OR 3.2: skin ulcer, gangrene, chronic wounds or deep tissue infection) [1], and in patients newly admitted to a geriatric unit in Belgium (OR 3.5: chronic wound) [52]. (Of note, the risk factor definitions in these latter studies were slightly different.) Also, the presence of an urinary tract catheter (UTC) was significantly associated with MRSA carrier status (OR 5.2). The risk is noticeably lower than in the Euregio study (OR 8.1) [44], but more than twice as high as the OR 2.2 in the AP study [1]. Other studies found an UTC only significant in univariate analysis (OR 2.8, LTCF 2011 in Belgium) [51]. Finally, a ‘history of multiple decolonisation cycles’ practically includes the risk factor ‘history of MRSA carriage’ (in this study in univariate analysis associated with highest risk factor for MRSA colonisation, as suggested previously [1, 44, 51–54]). History of MRSA carriage was also found the predominant risk factor in our AP study, being associated with a higher OR than in the LTCF population (univariate OR 9.9 vs. 7.6). However, to our knowledge this is the first time ‘history of multiple decolonisation cycles’ (multivariate analysis, OR 2.8) appearing as risk factor. Also in both studies, ‘contact with an MRSA carrier’ was significantly associated with MRSA carrier status, and—according to the univariate analysis—again, in the LTCF study this associated risk was higher than in the admission prevalence study (4.9 vs. 3.3). The latter observation is of interest as in LTCF studies this risk factor has previously only been rarely investigated or considered [1, 44]. Male gender appeared to be a risk factor for MRSA carriage only in the univariate analysis. This has been found to be a risk factor in previous MRSA prevalence studies [1, 38, 43, 51, 55], probably because of the higher frequencies of other factors predisposing men rather than women to MRSA acquisition [55]: In our study, male LTCF residents reported more frequently history of MRSA carriage, a urogenital infection, a urinary tract catheter, or a PEG tube—summarised under ‘impairment of the skin barrier‘–therefore, these factors may be confounders for the risk factor ‘male gender‘. Generally spoken, the older the resident, the rarer an MRSA colonisation was encountered in our study population. Looking at the group of male residents alone (significantly younger than the females (t-test, two-sided, p = 0.01)), the trend points in the opposite direction: the older the male LTCF residents are, the higher is the risk of colonisation. However, these results are not significant. Other studies could not identify the resident’s gender as a risk factor [33, 35, 37, 43, 56, 57].

In this study, a number of risk factors previously reported to be associated with MRSA carrier status were not found to be significant different between MRSA carriers and non-carriers. Diabetes has been a risk factor with a strong predictive value in previous studies [1, 54], but was confirmed neither in this nor in the Luxembourg study [43]. Also chronic dialysis, a risk factor in our AP study, was only reported in one of the MRSA carriers in this study. The use of antibiotics in the past three months—a significant risk factor in other studies [55, 58, 59]–could not be validated as a significant risk factor; this may confirm findings of our AP study or the Euregio study [1, 44].

Previously, it has been suspected that most LTCF residents acquire MRSA during a hospital stay and not in the LTCF [19], yet, in our study ‘hospitalisation’ was not associated with increased MRSA colonisation, a finding which contrasts with the results of other studies [1, 33, 37, 44, 51, 59, 60]. This raises the question if the occurrence of MRSA in LTCF is indeed the consequence of the occurrence of MRSA in hospitals, or if the introduction of MRSA occurs by other transmission pathways.

Many national and international recommendations for infection prevention clearly stipulate that among LTCF residents routine screening for MRSA is not indicated. However, based on our risk factor analysis, at least the number of residents to be screened and the resulting number of MRSA carriers can be calculated according to our multivariate model.

If a screening procedure would take into account the presence of the single risk factor associated with highest univariate OR, i.e. ‘history of MRSA carriage’, 4.5% of residents would be needed to be tested, and such test would result in the identification of 22.1% of MRSA positive residents (Table 4). In order to detect a larger proportion of MRSA-positive residents based upon several risk factors evaluated, also a significantly larger proportion of residents would be needed to be examined. In contrast to our AP study which allowed a risk-factor based detection of more than ⅔ of the MRSA carriers upon examining only ⅓ of the admission patients, in the LTCF resident population the risk factors are more numerous and therefore cover each a smaller part of the overall picture. Using the combination of the multivariate risk factors ‘ulcer / deep soft tissue infection’, ‘urinary tract catheter’, and ‘multiple decolonisation cycles’ with optimum outcome in the cost-benefit ratio, every eighth LTCF resident screened would be an MRSA carrier. However, only 38.2% of the total MRSA carriers would be detected at this point. Moreover, a screening policy based on the different risk factors according to RKI recommendations [30–32] (‘nursing home‘, ‘old age‘, and ‘dependency‘ as risk factors excluded) would also result in an impractically large proportion of residents to be screened (screening of 15–20 residents to detect one MRSA carrier). Thus, in the LTCF resident population the presence of select risk factors appears to be clearly less predictive for an MRSA carrier status when compared to acute care facility admission studies. In fact, if all MRSA carriers should be detected, all residents would have to be screened. However, our risk factor analysis reveals conditions which may facilitate the decision for active MRSA detection on individual indication (e.g. upon planned hospitalisation). In addition, not only transmission within the institution might be prevented by appropriate barrier precautions, also offering decolonisation to previously unknown MRSA carriers may protect them from infection by their own isolate.

Of our total of 136 MRSA carriers detected, history of MRSA carrier status based on a previous examination was known in only 30 carriers (22.1%). Moreover, according to the treatment history available, 17 of these 30 MRSA (56.7%) were reported to have undergone a complete and successful decolonisation. Therefore, a negative history or a negative status for MRSA colonisation did not preclude the presence of MRSA at the time of the prevalence study.

This study has potential limitations. The participation rate of 48% of the Saarland LTCF institutions at our study, and the participation rate of 68.8% of residents in the participating LTCF is not optimal, and we have no data on the biography or risk factors in non-participating institutions or residents. Thus, a selection bias cannot be excluded. Yet, in comparison to other comparable studies [34, 36, 38, 61], this participation rates appear to be among the highest being reported so far in this setting. Another limitation may be the omission an enrichment step during the laboratory analysis. In fact, in previous MRSA prevalence studies by other groups, broth enrichment procedures were used to enhance sensitivity, yet, those studies used conventional cotton swabs known to retain the microorganisms. However, with the use of flocked swabs (ESwabs) such enrichment step might be omitted. In a pre-study as part of the 'Methods' section of our AP study [1], comparable results were obtained when comparing the ESwab method and conventional swabs in conjunction with a broth enrichment step. These results have recently been supported by two additional studies [25, 26]. Thus, we conclude it unlikely that analytic sensitivity has been importantly diminished. We also purposely refrained from sampling chronic wound specimen, as in the AP study [1] the sole positivity of MRSA in a chronic wound was found to be exceedingly low (one patient with MRSA positivity only in the wound out of 20.027 participating patients). Moreover, during protocol establishing we were informed by LTCF personnel that the requirement of a wound swab in addition to the nasal and pharyngeal swab would likely have impaired the overall participation rate due to organisational constraints. This is also why some (potential) risk factors were not ascertained, e.g. the duration of residence in the LTCF. While we cannot rule out this factor as a potential contributor to the risk of MRSA acquisition, this item (as well as other potentially relevant items) was not included in the questionnaire in order to keep the questionnaire manageable for the care personnel documenting the various items.

The realisation of this study has been embedded in major information campaigns among Saarland nursing home personnel and administrators, but also among state legislators and the public, on the appropriate prevention measures for MRSA in elderly and nursing home facilities. Emphasis was put on any avoidance of care measures which could entail a consequence of social deprivation or interference with the daily life of the residents, while encouraging basic hygiene measures, appropriate contact prevention measures when performing transmission risk-associated care procedures, and introducing and teaching appropriate MRSA decolonisation protocols.

It has become clear that the overall awareness to control and prevent colonisation and infection with MRSA has state-wide increased, and it is hoped that this campaign contributes to improving the overall quality for LTCF residents towards their end of life.

In conclusion, in this study performed in an entire German Federal State the overall MRSA burden as well as the major risk factors for MRSA carrier status could be determined for a large number of LTCF. Related to the Saarland acute care hospital admission prevalence study [1], this study reveals that the MRSA prevalence among acute care admission patients is lower than the point prevalence among LTCF residents (2.2% vs. 4.8%). On the other hand, the prevalence of patients admitted to geriatric wards/departments in the AP study was 7.6%. This indicates that a patient population with an overall larger MRSA prevalence than the one determined in the overall LTCF resident population is admitted to acute care geriatrics departments. This LTCF study now provides a solid basis for estimating the burden of MRSA prevalence in these two major types of health institutions in one region; however, as this study does not reveal a single risk factor or a set of risk factors sufficiently predictive for an MRSA carrier status among LTCF residents, revealing the long term care setting being more complex. A risk factor based screening policy among LTCF residents therefore appears not to be justified.
